# Read's Case of Excision of Knee-Joint

**Published:** 1864-06

**Authors:** M. J. De Rosset

**Affiliations:** Assistant-Surgeon P. A. C. S.


ART. II.—*Read's Case of Excision of Knee-Joint.* Reported by M. J. DE ROSSET, Assistant-Surgeon P. A. C. S.

Captain C. K., 10th Louisiana infantry, aged 26 years, of good constitution, was wounded by a Minie ball, November 27, 1863, and, after some transportation by ambulance over rough roads and by rail, entered hospital November 30th. His condition on admission was good, both as regards the nervous and vascular systems. He presented a small circular wound on the outer aspect of right thigh, one and a half inches above the external condyle, on a plane somewhat posterior to it, and a clean incision, one and a half inches long, touching as a tangent the outer and upper edge of the patella, where, he says, the ball was cut out. There appears to have been no hemorrhage, but utter inability to walk. Ordered moist dressings.

December 2d.—A thin serous discharge appeared at the incised wound this morning. The movements of the knee-joint are perfect. Ordered a saline aperient, moist dressings and absolute rest.

3d.—Serous discharge continues in minute quantities, mingled with incipient suppuration. No inflammatory action.

8th.—Complains of considerable pain in the joint; slightly accelerated pulse (84); but all appearance of local inflammation is prevented by cold affusions. At Surgeon Read's suggestion, warm fomentations were substituted for the cold dressings. At 9 P. M., local pain, swelling, congestion and heat well marked; serous discharge greater at wound of exit. Explorations with finger and probe discovered no fracture, or loose bone. Pulse 104, full.

9th.—Pulse 128; local symptoms increasing, a consultation was called, and excision was decided upon. An elliptical incision was made, with its concavity upwards, around the patella, eight inches long, extending from over one condyle to the other, dividing the ligamentum patellæ. The joint was laid open; one and a half inches of the os femoris and half an inch of the tibia removed; the patella was uninjured, but was ablated also. No vessel required ligating.

The operation developed the following condition of things: There was a groove (as if it had been bored) extending from the external condyloid ridge of the femur to the superior edge of the cartilage covering the articular surface, and opening the synovial sac. The patella was untouched. The fluids in the sac escaped in large quantities, being reddish, thin, and containing clots of blood or shreds of fibrine, but no pus. The membrane itself was a little congested, but not discolored, and the parts adjacent and external to the joint extensively engorged with inflammatory exudation. This exudation had developed into good reparative material at the wound of exit, elsewhere it seemed to be degenerating into pus.

Silver sutures (two in number) were used to wire the bones, of which the adaptation was perfect; the entire wound was closed in like manner. (Surgeon Read operated, assisted by Surgeons Gibson and Michel and the hospital staff.)

A hastily-constructed box, similar to the one used by Mr. Butcher of London, was prepared to receive the limb, and a half grain of sulphate of morphia was administered.

10th.—Patient slept well. Pulse 124. Moist dressings.

11th.—Pulse 120. Ordered half an ounce of brandy every two hours, eggs, oysters and beef tea as diet. Suppuration commenced, probably thus early from the old exudation.

16th.—Patient has always been peevish, and his restlessness during the night produced some venous hemorrhage, (about six ounces,) easily controlled by injecting liquor ferri persulphatis. He naturally looks somewhat anæmic. Pulse 130.

18th.—Wound florid and healthy; some sutures were removed to facilitate the escape of pus. Pulse 110. Continue brandy, &c.

20th.—Slight hemorrhage during the night, (two ounces,) apparently from the granulations. A brisk cathartic was given, producing two evacuations. Complains greatly of the splint, which, from its hasty construction, is rather rude, and presses against both nates.

An apparatus, devised and adapted to this case, was then applied. It consists of three pieces. One a long posterior splint, extending from the upper third of the thigh to the superior border of the os calcis, narrow at the popliteal space, and supporting a movable foot-board, attached to the lower end by two rods; the whole being well padded to the sinuosities of the limb. Two side splints, interrupted at the knee and preserving their continuity by two iron angular brackets, projecting laterally two and a half inches, permitting of cleansing, &c. These extend to within one inch of the malleolus on each side, and are well padded. A suspensory apparatus of arbitrary device (either Smith's anterior-wire splint or the simple canvas bag above and below) may be applied, and the whole suspended to an arrangement resembling Salter's apparatus, but admitting of lateral motion, by means of rollers, which add materially to the ease of motion and comfort of the patient. To Acting Assistant Surgeon H. L. Thomas is due the credit of suggesting the use of the Smith wire splint, as a ready and efficient substitute for the iron brackets, and it is to be hoped that the success following this operation, the perfect security and convenience for dressings that this apparatus obtains, and the easy application of the wire splint, will induce surgeons in the field to weigh well the advantages of excision over amputation of the thigh in wounds of the knee-joint. The wire is to be bent into right angular recesses, at points corresponding to the knee, and wooden slats, padded, bound to them on the inner side, above and below.

22d.—The tumefaction bandage cuts the tendo-Achillis, and the whole is re-applied, a thin layer of cotton enveloping the foot and leg.

28th.—Has been comfortable since last report. To day, stomach is irritable from the administration of croton oil, which acted copiously. Pulse 98. Ordered synapisms to the epigastrium, ice, effervescing draughts and bismuth. The suture wiring the bone on the inner side came away this morning. Take half a grain of sulphate of morphia every night.

January 3d, 1864.—Internal half of incision closed. Appetite excellent, and is gratified with venison, partridges, turkey, &c. Is allowed one pint of porter daily.

7th.—Parts have a doughy sensation, receiving the impression of the fingers as in œdema. This is experienced more particularly on the outer aspect, extending from the angle of incision downwards to the sural muscles. Suppuration is healthy, and an increased flow can be produced by pressure along the tibia. Warm fomentations, with covering of oiled silk, were ordered.

10th.—Fomentations have been continuous, with marked subsidence of the swelling. No fluctuating point discovered. Bowels costive. Pulse 96. Ordered one ounce of Rochelle salts.

14th.—Wound healing from the external angle and from the apex, where pulsation may be observed communicated to the discharges by newly-formed vessels: Granulations florid, and contracting approximate lips of incision.

20th.—A few larvæ, deposited on the posterior splint by flies, necessitated the re-application of the splint, which was done without pain to the patient. Union, still of a yielding, flexible nature, has taken place between the bones; but no grating sound, nor any denuded bone, could be discovered with the probe.

28th.—Some indiscretion in diet has deranged the bowels and produced high fever. Pain in abdomen with borborigma Pulse 140. Ordered, calomel, four grains; ext. colocynth co, five grains; camphor, two grains—mix and take immediately. Liniment camphor. comp. to abdomen.

29th.—Much better. Prescription acted well. Pulse 100. Ordered one grain of pulv. opium every two hours.

February 14th.—Improving rapidly. A thin serous discharge has replaced the purulent. About half an inch still unhealed. Applied starch bandage, with aperture for wound.

25th.—Sits up daily. An effort was made to remove the remaining wire, but it could not be discovered. Two small spiculæ of bone were brought away, which did not appear to be necrosed.

March 15th.—Digestive apparatus is kept constantly deranged by capricious and enormous indulgence; he therefore requires frequent administration of opiates. Sits up daily, and has attempted to walk with crutches.

April 1st.—There is now slight lateral motion between the bones and very little antero-posteriorly; the leg is well supported by the reparative material, without assistance of splint or bandage, affording the deduction that well-organized fibrous tissue, of a ligamentous character, is at present the bond of union. Will not time convert this into bone? The natural size of the limb is regained, but a small opening still remains externally to the median line, from which issues, in minute quantities, a thin serous fluid, mingling with traces of pus gathered at the surface Measurement gives two and a quarter inches shortening for the affected limb. The foot has the natural eversion, and the limb, from trochanter to malleolus, is straight. A hardness and symmetrical roundness simulates the patella anteriorly.

Knowlton has been heard of to June 1—doing well and in excellent health.

